# T21. ALTERATIONS OF CRY2 AND PER3 GENE EXPRESSION ARE ASSOCIATED WITH GRAY MATTER ABNORMALITIES OF THALAMIC-LIMBIC NETWORK IN UNIPOLAR DEPRESSION AND BIPOLAR DEPRESSION

**DOI:** 10.1093/schbul/sby016.297

**Published:** 2018-04-01

**Authors:** Chengcheng Zhang, Peiyan Ni, Tao Li

**Affiliations:** West China Hospital, Sichuan University

## Abstract

**Background:**

The current study aimed to identify shared and distinct brain structure abnormalities and their relationships with circadian gene expression in patients with bipolar depression and unipolar major depression.

**Methods:**

A total of 103 subjects participated in this study, including 32 patients with bipolar depression (BDP), 26 patients with unipolar depression (UDP) and age, sex-matched 35 healthy controls were conducted brain structural magnetic resonance imaging scans and then used optimized voxel-based morphometry to explore group differences in regional gray matter volume (GMV). Circadian gene mRNA expressions in peripheral blood were measured on a subset of 12 patients with BDP, 19 patients with UDP and 33 control subjects using reverse transcription quantitative real-time polymerase chain reaction.

**Results:**

The GMV of the thalamus-limbic pathways had significantly increased in BDP cases relative to comparison subjects, while in UDP the increased GMV focused on the thalamus. The circadian-related gene mRNA expressions have significantly decreased in the patients with BDP, however with higher expression levels in the UDP cases. In addition, the GMV of right thalamus in the UDP was positively associated mRNA level of CRY2 gene, while the GMV of right hippocampus in the UDP was negatively associated mRNA level of PER3 gene.

**Discussion:**

Our study identified the relationship between abnormalities of thalamic-limbic network and alterations of circadian gene pathway in BDP and UDP. The shared GMV abnormality was the right thalamus. PER3 might be critical to hippocampus dysfunction in UDP, and CRY2 might be critical to thalamus dysfunction at a right-hemisphere function in BDP.

